# Impact of Simultaneous Nutrient Priming and Biopriming on Soybean Seed Quality and Health

**DOI:** 10.3390/plants13182557

**Published:** 2024-09-11

**Authors:** Gordana Tamindžić, Dragana Miljaković, Maja Ignjatov, Jegor Miladinović, Vuk Đorđević, Dragana Milošević, Dušica Jovičić, Slobodan Vlajić, Dragana Budakov, Mila Grahovac

**Affiliations:** 1 Institute of Field and Vegetable Crops, National Institute of the Republic of Serbia, 21000 Novi Sad, Serbia; dragana.bjelic@ifvcns.ns.ac.rs (D.M.); maja.ignjatov@ifvcns.ns.ac.rs (M.I.); jegor.miladinovic@ifvcns.ns.ac.rs (J.M.); vuk.djordjevic@ifvcns.ns.ac.rs (V.Đ.); dragana.milosevic@ifvcns.ns.ac.rs (D.M.); dusica.jovicic@ifvcns.ns.ac.rs (D.J.); slobodan.vlajic@ifvcns.ns.ac.rs (S.V.); 2 Faculty of Agriculture, University of Novi Sad, 21000 Novi Sad, Serbia; dragana.budakov@polj.edu.rs (D.B.); mila.grahovac@polj.edu.rs (M.G.)

**Keywords:** *Bacillus megaterium*, *Bradyrhizobium japonicum*, fungal infection, seed vigor and initial plant growth, soybean, Zn priming

## Abstract

In soybean production, numerous strategies are utilized to enhance seed quality and mitigate the effects of biotic and abiotic stressors. Zn-based nutrient priming has been shown to be effective for field crops, and biopriming is a strategy that is becoming increasingly important for sustainable agriculture. On the other hand, there is a lack of information about the effect of comprehensive nutrient priming and biopriming techniques on soybean seed quality and viability and seed health. This study was performed to assess the benefits of nutrient priming with Zn, biopriming with *Bacillus megaterium* and *Bradyrhizobium japonicum* (single and co-inoculation), and combination of nutrient priming and biopriming on the seed quality and viability, as well as seed infection caused by *Alternaria* spp. and *Fusarium* spp. Three different laboratory tests were employed: germination test, accelerated aging test, and seed health test. The results revealed that all tested priming treatments have a beneficial effect on seed germination, initial plant growth, and reduction of seed infection in normal and aged seeds. Additionally, comprehensive priming with Zn, *Bacillus megaterium*, and *Bradyrhizobium japonicum* reduced the occurrence of *Alternaria* spp. (−84% and −75%) and *Fusarium* spp. (−91% and −88%) on soybean seeds in the germination and accelerated aging tests, respectively, as compared to the control, which proved to be the most effective treatment in both optimal and stressful conditions.

## 1. Introduction

Soybean (*Glycine max* (L.) Merrill) is the most significant legume that is cultivated globally due to its edible beans that possess multiple uses. The importance of this staple crop lies in its ability to provide nitrogen fixation, high-quality protein, and oil [1]. It is observed that soybeans have the highest protein content, which ranges around 40%, while the oil content is around 20% [2], making them dominant in food and feed. Regarding food, the real value of soybean protein lies in its balanced amino acid profile, which corresponds with human dietary requirements [3]. In addition, all the essential amino acids such as tryptophan, valine, phenylalanine, histidine, isoleucine, leucine, lysine, methionine, and phenylalanine are contained in soybean plant protein that can adequately meet human physiological requirements that can adequately fulfill the physiological needs of humans [4,5]. Moreover, soybeans are the main source of proteins in animal feed. It has been estimated that approximately 68% of the soybean meal consumed in the European Union is used in the animal husbandry and poultry industries [6]. Soybeans are also known for their wide range of health benefits. The world’s soybean production is expanding annually due to all the advantages this crop offers. In this regard, it was observed that soybean production was +1286.94% higher in 2021 than in 1961, while the area harvested increased by +447.79% [7]. In Serbia, the harvested area has increased by 50.16% since 2016, with an upward trend [7].

A key factor in soybean production is prompt emergence and adequate seedling establishment. Additionally, seed germination and emergence are critical phases of plant development that significantly influence the subsequent stages of plant development in the field, indicating their economic and ecological significance [8]. As one of the most important stages in the existence of a plant, seed germination is a process that signals the transition from a quiescent to a metabolically active state and initiates a series of biophysical, biochemical, and molecular processes that ultimately result in the appearance of radicals and seedling growth [9].

Abiotic stressors such as drought, salinity, and extreme temperatures have a detrimental effect on seed germination by altering various internal metabolic and physiological processes. This occurs due to unfavorable conditions that reduce the seed’s absorption of water and energy and adversely affect the balance of hormones like gibberellins (GA) and abscisic acid (ABA), osmoprotectants like proline, reactive oxygen species (ROS), and the antioxidant defense system [9,10,11]. Abiotic stresses generally lead to reduced water supply to the seed or germinating seeds, resulting in slow metabolic processes and inhibiting or delaying germination [9]. Salinity causes serious stress, severely hindering seed germination and delaying seedling emergence [12]. It creates osmotic stress, which reduces the availability of water for the seeds, making the absorption process, which is crucial for seed germination, difficult. As a result, the seeds struggle to absorb water, leading to delayed germination and reduced germination rates [13,14]. It has also been shown that salinity affects hormonal balance within seeds [15]. Moreover, according to Tarnawa et al. [16], temperature has a significant impact on germination time and seedling development, while the temperature range between 15 and 20 °C is considered optimal. Additionally, both physiological and biochemical metabolic processes are significantly impacted by temperature [17]. The latter can control biochemical reactions and enzyme activity throughout the germination initiation phase. The metabolic activities required for germination and development are limited by low temperatures, which also inhibit food mobilization and diminish enzyme activity [18]. Moreover, seed-borne pathogens such as seed-borne fungi endanger plant productivity, affecting seed germination, viability, vigor, and initial seedling growth, thus affecting optimal plant population and good harvest, leading to a significant yield loss [19,20]. It has been reported that seed-borne fungi can lead to yield losses averaging around 9.4% globally due to their detrimental effects on seed quality and germination rates [21]. Overall, biotic and abiotic stressors are becoming more common as a result of climate change, posing a serious threat to plant productivity.

In recent years, seed enhancement techniques have emerged as essential tools for growing plants that can withstand a range of challenges. Among them, seed priming stands out as one of the most promising techniques since it treats seeds with natural and artificial compounds prior to germination, inducing a certain physiological state in plants. Seed priming involves the controlled hydration of seeds to activate germination processes without allowing emergence. Several types of seed priming techniques exist, each with unique benefits and procedures. Nutrient priming, i.e., pre-soaking with essential micronutrients, has been known for its beneficial effects on enhanced germination, improved seedling development, and increased stress tolerance in many plant species [22,23]. This technique provides seeds with the essential nutrients required during the crucial stage, thus leading to prompt and more uniform germination compared to unprimed seeds [23]. Enhancing crop emergence, seedling establishment, and seed metabolism can be achieved through seed priming with micronutrients such as zinc (Zn) and iron (Fe) [24,25,26]. Zn is an essential nutrient that is one of the constituents of over 300 enzymes and is the sole component present in each of the six groups of enzymes [27]. It is also well-known for its bio-physicochemical functions in plants, which include protein synthesis, carbohydrate metabolism, gene regulation and activation, and morphological and anatomical roles in bio-membranes [27,28,29]. Seeds with elevated Zn content have the ability to withstand unfavorable conditions, produce normal seedlings, and, thus, ensure higher yields [30,31,32]. Moreover, Zn enhances plant tolerance through involvement in the detoxification of ROS such as O_2_−(superoxide radical) and H_2_O_2_ (hydrogen peroxide), and it plays a direct role in protein synthesis generally, auxin biosynthesis [33], gibberellins [34], plant growth regulation, and membrane stability [35,36]. Also, micronutrient-primed seeds, such as zinc (Zn), can strengthen their resistance against soil-borne pathogens [34]. Higher concentrations of these nutrients in the seeds can improve germination and seedling vigor, potentially making plants less susceptible to fungal infections during the early growth stages [37].

Moreover, biopriming is a beneficial, eco-friendly priming technique that involves the application of beneficial microorganisms or their products through priming and, as such, is increasingly important in sustainable agriculture. It has been proven to have significant effects of biopriming on the speed and uniformity of seed germination, crop establishment, and yield of soybeans [38,39,40]. Furthermore, one of the major advantages of biopriming is its role in enhancing plant resilience against biotic (pests and diseases) and abiotic (drought, salinity, temperature) stresses [41]. By stimulating the production of protective metabolites and enhancing the synthesis of stress-related proteins, bioprimed seeds exhibit improved tolerance to adverse environmental conditions, which is achieved through various biochemical and molecular mechanisms that bolster the plant’s defense systems [41,42]. Beneficial rhizobacteria, characterized as plant growth-promoting rhizobacteria (PGPR), have been reported to benefit plant growth and development under various stresses by producing plant hormones, increasing nutrient availability, and suppressing plant pathogens [39]. In spite of our current understanding of the aforementioned facts, data is scarce regarding comprehensive soybean seed priming with Zn and PGPR, such as *Bacillus megaterium* and *Bradyrhizobium japonicum*. Particularly, there is a lack of information on the effect of these seed priming techniques and their combination on soybean resilience to seed-borne fungal infections. Therefore, the objective of this study was to assess the effects of nutrient priming and biopriming, as well as their combination, on seed germination, initial seedling growth, and seed health of soybean under optimal and stressful laboratory conditions.

## 2. Results

Table 1 presents the effects of seed priming treatments, nutrient priming and biopriming, and their interaction. The germination test results demonstrated that nutrient priming and biopriming significantly affected all examined soybean parameters. Factor nutrient priming (NP) had a significant effect on all soybean parameters as well as factor biopriming (BP). Nutrient priming and biopriming interaction also significantly altered all examined parameters of the soybean cultivar, except for the presence of *Fusarium* spp. Furthermore, in the accelerated aging test, results showed that both factors and their interaction have significant effects on all examined parameters as well, except for the presence of *Alternaria* spp. and *Fusarium* spp.

### 2.1. The Germination Test

Generally, the soybean cultivar responded to nutrient priming and biopriming treatments regarding seed germination and initial plant growth (Table 2; Supplementary Materials, Table S1). The results showed that Zn priming had a significant effect on seed germination on average, while the most favorable impact on this parameter was biopriming with B. megaterium on average, where the highest seed germination was observed in the treatment with Zn and B. megaterium (94.25%) compared to the control (84.75%) (Table 2; Supplementary Materials, Table S1). On average, biopriming with *Br. japonicum* and *B. megaterium* + *Br. japonicum* also had beneficial effects on seed germination compared to the control, but to a lesser extent. Regarding the abnormal seedlings, on average, no significant differences were recorded in hydropriming and Zn priming compared to the control. At the same time, biopriming with *B. megaterium* + *Br. japonicum* followed by priming with *B. megaterium* significantly reduced the percentage of abnormal seedlings (Supplementary Materials, Table S1). Within hydropriming and Zn priming, biopriming with *B. megaterium* + *Br. japonicum* reduced the occurrence of abnormal seedlings by +47.3% and +32.1%, respectively, as compared to the control.

Moreover, the examined treatments also had a favorable effect on initial plant growth (Table 2; Supplementary Materials, Table S1). On average, Zn priming had the best impact on shoot and root length, followed by hydropriming. Regarding the effect of biopriming treatments, no difference was observed between particular treatments, and all of them had a significant effect on shoot length compared to the control. The highest shoot length was observed in comprehensive treatment with Zn and B. megaterium (152.9 mm), followed by Zn and *B. megaterium* + *Br. japonicum* (151.8 mm) (Table 2; Supplementary Materials, Table S1). Additionally, on average, all biopriming treatments led to a significant increase in root length; the greatest effect was observed in biopriming with *B. megaterium*, followed by *B. megaterium* + *Br. japonicum* and *Br. japonicum*. The highest values of root length were observed in comprehensive treatment with *B. megaterium* and Zn priming (163.5 mm), followed by *B. megaterium* and hydropriming (157.0 mm) compared to the respective control.

In addition, the examined treatments also had a beneficial effect on soybean biomass accumulation under optimal conditions (Table 3; Supplementary Materials, Table S1). It was observed that Zn priming, followed by hydropriming, significantly improved the fresh and dry biomass accumulation of soybean seedlings compared to the control (Supplementary Materials, Table S1). Regarding the biopriming treatment, a similar pattern of biopriming treatments was observed in fresh and dry shoot and root weight. The highest increase in fresh and dry shoot weight was observed in biopriming with *Br. japonicum* and *B. megaterium* + *Br. japonicum*, followed by *B. megaterium*, while for fresh and dry root weight, the most significant increase was observed in biopriming with *Br. japonicum*, followed by *B. megaterium* + *Br. japonicum* and *B. megaterium*. The highest values of fresh and dry shoot biomass accumulation were observed in comprehensive treatments with Zn and *B. megaterium* + *Br. japonicum* (11.07 g and 1.169 g, respectively), followed by Zn priming and biopriming with *Br. japonicum* (11.05 g and 1.157 g, respectively) (Table 3; Supplementary Materials, Table S1). For fresh and dry root weight, the most beneficial impact was observed in the comprehensive treatment with Zn and *Br. japonicum* (2.50 g and 0.241 g, respectively) compared to the control (Table 3).

Likewise, the seedling vigor index was also impacted by priming treatments (Table 3, Supplementary Materials, Table S1). On average, Zn priming significantly improved the seedling vigor index, followed by hydropriming. For biopriming treatments, a similar pattern has been observed for seed gemination; the most beneficial effect was observed for treatment with *B. megaterium*, followed by *B. megaterium* + *Br. japonicum* and *Br. japonicum* (Supplementary Materials, Table S1). However, regarding the seed vigor index, soybean reacted differently to biopriming treatments within the nutrient priming treatments; in control, the highest SVI was recorded in priming with *B. megaterium* + *Br. japonicum* (2604.9), while in hydropriming and Zn priming, biopriming with *B. megaterium* led to the highest increase of SVI (2743.4 and 2982.2, respectively) compared to the control.

### 2.2. Accelerated Aging Test

To assess the seed viability, an accelerated aging test was performed. The obtained results showed that, on average, Zn priming did not improve seed germination of soybean-aged seeds, while hydropriming led to a decrease in seed germination compared to the control (Table 4; Supplementary Materials, Table S2). Contrary to this, priming with *B. megaterium* + *Br. japonicum* (+4.61%), followed by priming with *B. megaterium* (+3.04%), significantly improved soybean seed germination, while treatment with *Br. japonicum* led to a decrease of seed germination (−2.51%) compared to the control (Supplementary Materials, Table S2). Within control and Zn priming, priming with *B. megaterium* and *B. megaterium* + *Br. japonicum* (+5.69% and +4.67%, respectively) significantly improved seed germination compared to the control. Contrary to this, within hydropriming and Zn priming, priming with *Br. japonicum* led to a decrease in seed germination of aged soybean seeds (−3.77% and −3.43%, respectively). Moreover, on average, hydropriming led to a decrease in the percentage of abnormal seedlings, while no significant differences were observed in biopriming treatments compared to the control (Supplementary Table S2). Within control, priming with *B. megaterium* significantly decreased the percentage of abnormal seedlings by −18.3%, while, on the contrary, treatment with *Br. japonicum* led to an increase of abnormal seedlings (+32.7%). However, within hydropriming and Zn priming, no significant differences were observed (Table 4).

Regarding the initial plant growth, it was observed that hydropriming followed by Zn priming significantly reduced shoot length of aged soybean seeds (Table 4; Supplementary Materials, Table S2). However, biopriming treatments significantly improved shoot length of the aged soybean seeds; the greatest effects were observed in priming with *B. megaterium*, followed by *B. megaterium* + *Br. japonicum* and *Br. japonicum*. The highest shoot length, however, was recorded when aged seeds were only bioprimed with *B. megaterium* + *Br. japonicum* (150.50 mm) (Table 4). Moreover, root length of the aged soybean seeds gradually decreased with hydropriming and Zn priming compared to the control, while all examined biopriming treatments significantly improved this parameter (Table 4; Supplementary Materials, Table S2). In this regard, biopriming with *B. megaterium* had the greatest effect on root length compared to the control, followed by *B. megaterium* + *Br. japonicum* and *Br. japonicum* (Supplementary Materials, Table S2). Also, within the control, hydropriming and Zn priming, priming with *B. megaterium* proved to have the most beneficial effect on root length of the aged soybean seeds (+135.6%, +65.5%, and +46.1%, respectively) (Table 4).

Regarding the biomass accumulation, it was observed that, on average, hydropriming and Zn priming led to an increase in fresh and dry shoot weight and a decrease in fresh and dry root weight (Table 5; Supplementary Materials, Table S2). Likewise, biopriming with *Br. japonicum* followed by *B. megaterium* + *Br. japonicum* had a beneficial effect on the fresh and dry shoot biomass of aged soybean seeds, while, for fresh and dry root weight, all examined treatments had a positive effect; however, among them, biopriming with *Br. japonicum* stood out as the most efficient compared to the control on average (Supplementary Materials, Table S2). In control, only treatment with *B. megaterium* + *Br. japonicum* improved fresh shoot weight (9.80 g); in hydropriming, *B. megaterium* + *Br. japonicum* and *Br. japonicum* proved to be the most effective (10.08 g and 9.98 g), while comprehensive priming with Zn and Br. japonicum (10.58 g) led to the highest increase in fresh shoot weight. Contrary, it was recorded that comprehensive priming with Zn and *B. megaterium* + *Br. japonicum* led to a decrease in the fresh shoot weight of aged soybean seeds by −3.9%. As for fresh root weight, all comprehensive priming treatments significantly improved the fresh root weight of aged seeds. The decrease in dry shoot weight was observed in the following treatments: *B. megaterium* and *Br. japonicum* in control; *B. megaterium* and hydropriming; *B. megaterium* + *Br. japonicum* in Zn priming. Contrary to this, comprehensive priming treatments B. megaterium and Zn priming (0.952 g) and *Br. japonicum* and Zn priming (1.096 g) significantly improved dry shoot length of aged seeds. As for the dry root weight, all comprehensive priming treatments had favorable effects compared to the respective controls (Table 5).

In addition, the effect of different priming treatments on seedling vigor index was also examined (Table 5; Supplementary Materials, Table S2). Thus, it was observed that, on average, seedling vigor index was decreased by Zn priming followed by hydropriming, while all examined biopriming treatments led to an increase in this parameter (Supplementary Materials, Table S2). The highest values of seedling vigor index were recorded in the following treatments: *B. megaterium* in control (2709.2), *B. megaterium* and hydropriming (2130.4), *B. megaterium* + *Br. japonicum* and Zn priming (2364.7) compared to the respective control (Table 5). The only decrease in seedling vigor index was observed in comprehensive treatment *Br. japonicum* and Zn priming (1375.7) compared to the control (1600.0).

According to the results of Pearson’s correlation analysis, examined parameters were significantly correlated in both laboratory tests, except for abnormal seedlings and dry root weight in the germination test, and abnormal seedlings and other parameters as well as fresh root weight versus fresh shoot weight and dry shoot weight in the accelerated aging test (Table 6). In the germination test, seed germination of soybean seeds was highly correlated with shoot and root length, fresh and dry shoot weight, seedling vigor index (*p* < 0.001), and fresh and dry root weight (*p* < 0.01), while a significant negative correlation was observed between seed germination and abnormal seedlings (*p* < 0.01) (Table 6a). A negative interrelationship was established between abnormal seedlings and shoot and root length, fresh and dry shoot weight, and seedling vigor index. Moreover, shoot length was significantly correlated with root length, fresh and dry shoot and root weight, and vigor index. The positive correlation was also established between root length and fresh and dry shoot and root weight, and vigor index; fresh shoot weight and dry shoot weight, fresh and dry root weight, and seedling vigor index; fresh root weight and dry shoot and root weight, and seedling vigor index; dry shoot weight and dry root weight and seedling vigor index; dry root weight and seedling vigor index.

In the accelerated aging test, a positive interrelationship was observed between seed germination and shoot and root length, fresh and dry root weight, and seedling vigor index; a negative interrelationship was established between seed germination and fresh and dry shoot weight, while no interrelationship was established between seed germination and abnormal seedlings (Table 6b). Abnormal seedlings did not correlate with other examined parameters of aged soybean seeds. A positive correlation was established between shoot length and root length, fresh and dry root weight, and seedling vigor index, while a negative correlation was established between shoot length and fresh and dry shoot weight. Root length was positively correlated with fresh and dry root weight and seedling vigor index, while a negative correlation was recorded between root length and fresh and dry shoot weight. Moreover, a positive correlation was observed between fresh shoot weight and dry shoot weight, while a negative correlation was observed between fresh shoot weight and fresh and dry root weight and seedling vigor index. Fresh root weight was positively correlated with dry root weight and seedling vigor index, while a negative interrelationship was established between fresh root weight and dry shoot weight. However, a negative interrelationship was observed between dry shoot weight and dry root weight and the seedling vigor index. Contrary to this, dry root weight was positively correlated with seedling vigor index.

### 2.3. Seed Health

The blotter method was employed to evaluate the health of the seeds under optimal and stressed conditions of an accelerated aging test, and the obtained results are presented in Table 7 and Table 8 and Supplementary Materials Table S3.

Under optimal conditions, the results revealed that, on average, Zn priming significantly reduced the occurrence of *Alternaria* spp. and *Fusarium* spp., while, in terms of biopriming treatments, all examined biopriming treatments reduced the presence of *Alternaria* spp., and treatments *B. megaterium* and *B. megaterium* + *Br. japonicum* were the most effective compared to the control (Supplementary Materials, Table S3a). Biopriming with *B. megaterium* decreased the occurrence of *Alternaria* spp. by −72.0% in control, −75.0% in hydropriming, and −71.4% in Zn priming, while priming with *B. megaterium* + *Br. japonicum* suppressed the presence of these pathogen fungi by −60.0%, −66.7%, and −71.4%, respectively (Table 7). Priming with *Br. japonicum* and hydropriming reduced the presence of *Alternia* spp. compared to the control, but to a lesser extent. Moreover, seed infection caused by *Fusarium* spp. was significantly suppressed by *B. megaterium* and *B. megaterium* + *Br. japonicum* treatments in control, hydropriming, and Zn priming compared to the respective controls (Table 7).

Regarding the aged soybean seeds, the results showed that, on average, Zn priming had a significant effect on suppressing both *Alternaria* spp. and *Fusarium* spp. At the same time, hydropriming reduced only the occurrence of *Fusarium* spp. (Supplementary Materials, Table S3b). Moreover, the positive effect of priming with *B. megaterium* and *B. megaterium* + *Br. japonicum* on the reduction of the presence of *Alternaria* spp. was observed, while all examined biopriming treatments significantly reduced the presence of *Fusarium* spp. (Supplementary Materials, Table S3b). Within the nutrient priming, priming with *B. megaterium* + *Br. japonicum*, followed by *B. megaterium,* proved to be the most efficient treatment in suppressing *Alternaria* spp. compared to the control (Table 8). The highest suppression of *Alternaria* spp. was observed in treatments Zn priming and *B. megaterium* + *Br. japonicum* (−66.7%). In terms of *Fusarium* spp., all examined biopriming treatments significantly suppressed this pathogen in comparison to the control (Table 8). However, Zn priming along with *B. megaterium* + *Br. japonicum* proved to be the most effective in controlling *Fusarium* spp. (−75%) compared to the control.

## 3. Discussion

In this study, it has been proven that Zn priming has a beneficial effect on soybean seed germination under optimal conditions. Regarding nutrient priming such as Zn priming, it has been proven that Zn has a favorable impact on seed germination and quality of maize [25,43,44,45], wheat [46,47], chickpea [47], soybean [48], rice [49], and other crops. It has been established that Zn, through the priming technique, enhances seed quality performance due to their involvement in seed metabolism at the initial phase of germination [50]. However, our results revealed that Zn priming did not improve the seed germination of aged soybean seeds. Similar results were also obtained in maize [43]. As stated by Varier et al. [51], highly vigorous seed lots are threatened after priming due to seed decay, where the most common cause is damage to membranes and other subcellular components caused by harmful free radicals caused by the peroxidation of unsaturated and polyunsaturated fatty acids in the membrane. Generally, seed priming improves the longevity of low-vigor seeds, while longevity decreases in high-vigor seeds [43,52]. Moreover, these results are consistent with the findings of Girolamo and Barbanti [53], who stated that the sucrose and raffinose family oligosaccharides (RFOs) play an important role in desiccation tolerance and seed longevity, and a lack of their accumulation during the drying-back process in the priming technique is responsible for a reduced formation of the glassy layer at the membrane level, resulting in accelerated deterioration [54].

On the other hand, biopriming with *B. megaterium* and *Br. japonicum*, as well as their combination, had a significant impact on soybean seed germination as well. The beneficial effects of these plant growth-promoting bacteria were also established on two soybean cultivars under optimal and stressful conditions [39]. It is found that these microbials have the ability to produce indole compounds like indole acetic acid (IAA), which stimulate cell division and the growth of the embryo, promoting germination [55]. Furthermore, our results also revealed that the most favorable effect on seed germination is the simultaneous, comprehensive priming with Zn and *B. megaterium* under optimal conditions. According to earlier studies, *Bacillus* spp. possess a multitude of PGP traits, including Zn solubilization by the secretion of organic acids, such as 2-ketogluconic acid and gluconic acid, proton extrusion, and production of chelating ligands [56,57]. Also, it has been demonstrated that zinc oxide nanoparticles (ZnO NPs) can be produced extracellularly via the secondary metabolites and enzymes of *Bacillus* strains [58,59]. However, it was found that only biopriming has beneficial effects on the seed germination of aged soybean seeds, with the prevalence of combined *B. megaterium* and *Br. japonicum*, as well as *B. megaterium*, being the most effective. These results confirm previous findings indicating that these bacteria are good biofertilizers and growth promoters, which have a positive effect on the seed quality of many crops [39,60,61,62].

Likewise, the results of this study also showed that Zn priming significantly improved initial plant growth and biomass accumulation under optimal conditions, while the positive effect was lacking under unfavorable conditions of the accelerated aging test. Zn has a significant impact on the accumulation of essential proteins and is important in many physiological and biochemical processes such as chlorophyll biosynthesis, photosynthesis, respiration, hormone regulation, gene expression regulation, and environmental stress tolerance [35,63]. However, protein denaturations due to the high temperature and high relative humidity of the accelerated aging test were severely harmful to soybean seeds, leading to loss of biological activity, seed germination, and nutritional quality [64,65]. Furthermore, the particular context of accelerated aging testing implies that the harm caused by harsh conditions may overwhelm the protective advantages of Zn priming, even though Zn is known to have a role in different physiological systems and can aid in stress responses [65,66,67]. This suggests that Zn treatment may not substantially impact the repair processes in situations of extreme stress, such as high humidity and temperature.

Moreover, examined biopriming treatments also had a beneficial effect on initial plant growth and biomass accumulation under optimal and stressful conditions, where biopriming with *B. megaterium* stands out as the most beneficial under optimal conditions and biopriming with *B. megaterium* and *Br. japonicum* proved to be the most effective on soybean-aged seeds. The favorable effect of examined biopriming treatments can be explained by the fact that *B. megaterium* impacts plant growth through various mechanisms such as phytohormone production, nutritional enhancement, stress resistance, root architecture, and overall plant growth since it increases biomass production [68,69,70,71]. In addition, the simultaneous effects of Zn and *B. megaterium* proved to be the most effective regarding plant growth and development under optimal conditions. It is well known that *B. megaterium* may solubilize zinc from insoluble forms, increasing its availability to plants by chelating agents and organic acid formation, and the application of *B. megaterium* alongside zinc resulted in higher proximate constituents such as crude fiber, crude protein, and essential minerals [70,72]. Generally, the results showed that Zn priming improved seedling vigor index under optimal conditions, while biopriming proved to have a favorable effect on seedling vigor index in both conditions. These results are in agreement with the findings of Miljakovic et al. [39] and Tamindzic et al. [25].

The correlation analysis verified the favorable effects of seed priming treatments. Overall, a positive interrelationship was established between germination and other examined parameters, and, therefore, the examined nutrient priming and biopriming treatments could result in enhanced seed quality and vigor in soybean.

In addition, the assessment of seed health revealed that Zn priming significantly suppressed the presence of fungal pathogens, *Alternaria* spp. and *Fusarium* spp., which corroborates previous findings [73,74,75]. Zinc has a role in initiating multiple enzyme systems that control distinct physiological processes as well as defense mechanisms in plant-pathogen interactions [76]. Furthermore, Zn improves cell integrity and cell membrane stability, as well as increases plant vigor and root growth, which act as defense factors against fungal infection [73,77]. Also, it has been found that an adequate concentration of Zn improved plants defense response against *Alternaria brassicicola* through an enhanced JA/ET-dependent defense signaling pathway in *Arabidopsis thaliana* [76,78]. Regarding the beneficial effect of biopriming with *Bacillus* spp. on fungal infection, similar results were obtained in wheat [79], tomato [80], and peas [60]. Significant potential of *Bacillus* species in the fungal infection management induced by *Alternaria* and *Fusarium* species has been demonstrated [60,81]. Based on previous research, it can be concluded that certain *Bacillus* strains are efficient biocontrol agents since they display antagonistic capabilities against these harmful fungi [60,80,82,83]. In this regard, several mechanisms, such as the synthesis of antibiotics, hydrolytic enzymes, and siderophores, which aid in nutrient competition and pathogen inhibition, are used by *Bacillus* species to carry out their biocontrol activities [83,84]. Additionally, they may trigger plants to acquire induced systemic resistance (ISR), strengthening their defenses against infections [83,84]. The findings additionally demonstrated that *Bradyrhizobium japonicum* and *Bacillus megaterium* priming significantly reduce fungal infections caused by *Alternaria* spp. and *Fusarium* spp. Iturralde et al. [85] showed that *Br. japonicum* in co-inoculation with beneficial fungi, such as *Trichoderma harzianum*, may enhance plant resistance to fungal infections by promoting overall plant health and growth. Moreover, it was observed that *Br. japonicum*, togehter with nutrients, effectively colonizes root tips and enhances plant growth, which can indirectly help in suppressing fungal infections by promoting healthier plants that are more resilient to diseases [86].

Overall, these results indicate that comprehensive nutrient (Zn) and biopriming (*B. megaterium* and *Br. japonicum*), especially priming with *B. megaterium*, could enhance soybean seed quality and initial plant growth and could improve resilience to fungal infections such as *Alternaria* spp. and *Fusarium* spp. This comprehensive seed priming method can be suggested as a sustainable method to enhance seed quality and health. The soybean varieties and soil bacteria (*Br. japonicum* and *B. megaterium*) used for seed biopriming experiments will be further tested in the Serbian Living Lab within the VALERECO project (Horizon Europe project 101135472).

## 4. Materials and Methods

### 4.1. Plant Material

Seeds of the soybean cultivar NS Atlas were acquired from the Legume Department, Institute of Field and Vegetable Crops, National Institute of the Republic of Serbia, Novi Sad. Serbia. The NS Atlas is an early variety that belongs to Group 0. It is known for its high and consistent yield potential, exceeding 5.5 tons per hectare. This variety is of average height and overgrown with gray hairs, and its grain is of medium size, yellow color, and gray hilum. This variety is suitable for growing in hilly regions and is suitable for regular, late, and post-small-grain sowing.

### 4.2. Priming Treatments and Seed Priming

In the current study, the simultaneous effects of two different types of seed priming, i.e., nutrient priming and biopriming, on seed quality performance, seed vigor, and seed health of soybean under different laboratory conditions were assessed.

As for nutrient priming, seed priming with zinc was performed using Zinc Sulfate Heptahydrate (ZnSO_4_ × 7H_2_O) (Sigma Aldrich, St. Louis, MO, USA). The seeds of the selected soybean cultivar were primed in a 0.02% aqueous zinc sulfate solution at 25 °C for 4 h under dark conditions, as prescribed by Aboutalebian et al. [87]. Soybean seeds were also primed with distilled water as a positive control, whereas non-primed seeds were used as a negative control.

*Bacillus megaterium* and *Bradyrhizobium japonicum* strains from the Collection of the Laboratory for Microbiological Research (Institute of Field and Vegetable Crops, Novi Sad, Serbia) were utilized as biopriming agents. Bacterial culture suspensions were prepared and applied to soybean seeds, as described by Miljaković et al. [39]. *Bradyrhizobium* and *Bacillus* strains were cultured in yeast extract mannitol broth (YEMB) and nutrient broth (NB), respectively, and incubated at 28 ± 2 °C and 120 rpm (Edmund Bühler SM-30 B, Bodelshausen, Germany) for 72 h (*Bradyrhizobium* strains) and 24 h (*Bacillus* strains). The inoculum rate was adjusted to 2 × 10^9^ CFUs/g. Non-primed seeds were used as a control. Seed biopriming was performed using bacterial suspension at 25 °C for 5 h under dark conditions.

Following nutrient priming and biopriming, seeds were rinsed with distilled water and dried at room temperature for 72 h on sterile filter paper [39].

### 4.3. Laboratory Assays

To assess the seed quality and vigor as well as seed health, three laboratory tests were performed. The experiments were performed at the Laboratory for Seed Testing, Institute of Field and Vegetable Crops (IFVCNS), Serbia.

#### 4.3.1. Seed Quality Assessment

The germination test was employed to assess the primed seed quality of soybean seeds. The seed sample consisted of three replicates, with 100 randomly selected seeds per replicate. Each replicate (100 seeds) was sown in a plastic box (240 × 150 mm) filled with optimally moistened, sterilized sand as a substrate. The samples were placed in the germination chamber (Conviron CMP 4030, Winnipeg, MB, Canada) at a temperature of 25 °C with a day/night regime of 16/8 h for 8 days, as prescribed by ISTA [88]. The seed germination and abnormal seedlings were assessed on the 8th day post-sowing, following the prescribed protocol by the ISTA Rules for Seed Testing [88].

#### 4.3.2. Seed Vigor Assessment

To assess the seed vigor of soybean, the accelerated aging test was employed. The accelerated aging test was performed by placing seeds in a water bath (Vims-elektrik, WKP-14, Tršić, Serbia) with nearly 95% relative humidity at a temperature of 42 °C for 72 h [89]. Thereafter, the germination test was performed as described in Section 4.3.1 [88].

#### 4.3.3. Assessment of Germination-Related and Growth-Related Parameters of Soybean Seeds

The seedling vigor index (SVI) was determined as prescribed by Abdul-Baki and Anderson [90] using the following formula:

SVI = Seedling Length (cm) × Seed Germination (%)

The shoot and root length of ten normal seedlings, as well as fresh seedlings’ (shoot and root) weight, were determined on the same day as seed germination and abnormal seedlings [91,92]. To assess the dry shoot and root weight, seedlings were oven-dried (Heraeus instruments, Hanau, Germany) at a temperature of 80 °C for 24 h, after which the weight was obtained using a laboratory balance (Kern 770-13, KERN & Sohn GmbH, Balingen, Germany).

#### 4.3.4. Seed Health Assessment

Seed health testing was performed using the blotter method as described by Mathur and Kongsdal [93]. Four hundred seeds of each soybean seed sample were sterilized with 3% NaOCl (Sigma Aldrich, St. Louis, MO, USA) for 30 s and rinsed three times with sterile distilled water. The seeds were incubated for 7 days at 22 ± 2 °C with alternating cycles of 12 h light and 12 h darkness. The presence of a fungal infection was determined, and the results were presented in percentage.

Further isolation of pathogens was carried out by cutting a small piece of infected seed, which was surface sterilized with 3% NaOCl (Sigma Aldrich, St. Louis, MO, USA) for 3 min, dried and transferred onto potato dextrose agar (PDA) (Torlak-Institute for Virology, Vaccines, and Serums, Belgrade, Serbia), and then incubated for 7 days at 25 °C [94].

### 4.4. Statistical Analysis

A completely randomized design (CRD) with three replications was employed for the trials. The data obtained were statistically analyzed using ANOVA, and afterwards, mean separation was obtained by Duncan’s multiple range test (DMRT) (*p* ≤ 0.05). Seed health data were arc-sine square root transformed prior to ANOVA. Pearson’s correlation analysis was used to determine the relationship between the parameters. The STATISTICA 10.0 software (StatSoft Inc., Tulsa, OK, USA) was used to process the data statistically.

## 5. Conclusions

This study confirmed that nutrient priming with Zn and biopriming with *B. megaterium* and *Br. japonicum*, alone or in combination, have great potential for improving the seed quality, health, and viability of soybean seeds. Comprehensive priming with Zn and *B. megaterium* proved to be the most effective in the germination test and accelerated aging test, as well as the most beneficial in achieving soybean resilience to *Fusarium* spp. and *Alternaria* spp. fungal infections. This comprehensive seed priming method can be suggested as a sustainable method to improve seed germination and inital seedling growth, along with the suppression of fungal infection. Further research on effective, comprehensive priming treatments through field experiments will be needed to determine their efficacy under different conditions. Also, future studies should consider appropriate formulations and methods of application to ensure the desired efficacy of nutrient priming and biopriming under environmental conditions.

## Figures and Tables

**Table 1:** Analysis of variance for the examined parameters of soybean after seed nutrient priming and biopriming in the (**a**) germination test and the (**b**) accelerated aging test.

**(a) Germination Test**			
**Parameters**	**Nutrient Priming (NP)**	**Biopriming (BP)**	**NP × BP**
Seed Germination	0.0002 ***	0.0000 ***	0.1223 *
Abnormal Seedlings	0.1480 *	0.0000 ***	0.0004 ***
Shoot Length	0.0000 ***	0.0000 ***	0.0001 ***
Root Length	0.0000 ***	0.0000 ***	0.0000 ***
Fresh Shoot Weight	0.0000 ***	0.0000 ***	0.0000 ***
Fresh Root Weight	0.0000 ***	0.0000 ***	0.0000 ***
Dry Shoot Weight	0.0000 ***	0.0000 ***	0.0000 ***
Dry Root Weight	0.0000 ***	0.0000 ***	0.0000 ***
Seedling Vigor Index	0.0000 ***	0.0000 ***	0.0000 ***
*Alternaria* spp.	0.0000 ***	0.0000 ***	0.0009 ***
*Fusarium* spp.	0.0000 ***	0.0000 ***	0.0891 ^ns^
**(b) Accelerated Aging Test**			
**Parameters**	**Nutrient Priming (NP)**	**Biopriming (BP)**	**NP × BP**
Seed Germination	0.0003 ***	0.0000 ***	0.0332 *
Abnormal Seedlings	0.0175 *	0.3267 *	0.0016 **
Shoot Length	0.0000 ***	0.0000 ***	0.0000 ***
Root Length	0.0000 ***	0.0000 ***	0.0000 ***
Fresh Shoot Weight	0.0000 ***	0.0000 ***	0.0000 ***
Fresh Root Weight	0.0000 ***	0.0000 ***	0.0000 ***
Dry Shoot Weight	0.0000 ***	0.0000 ***	0.0000 ***
Dry Root Weight	0.0000 ***	0.0000 ***	0.0000 ***
Seedling Vigor Index	0.0000 ***	0.0000 ***	0.0000 ***
*Alternaria* spp.	0.0005 ***	0.0000 ***	0.7627 ^ns^
*Fusarium* spp.	0.0000 ***	0.0000 ***	0.8978 ^ns^

* *p* ≤ 0.05, ** *p* ≤ 0.01, *** *p* ≤ 0.001, ns—non-significant.

**Table 2:** The effects of priming treatments on seed germination, abnormal seedlings, and root and shoot length of soybean in the germination test.

Nutrient Priming	Biopriming	Seed Germination (%)	Abnormal Seedlings (%)	Shoot Length (mm)	Root Length (mm)
Control	Control	84.75 ± 1.1 b	9.50 ± 0.29 a	116.5 ± 0.46 c	131.1 ± 0.72 c
	Bac	91.00 ± 0.65 a	6.25 ± 0.65 bc	137.4 ± 0.38 ab	135.5 ± 0.54 b
	Bj	86.50 ± 0.91 b	7.50 ± 0.25 b	138.4 ± 1.00 a	133.0 ± 1.13 c
	Bac + Bj	90.75 ± 1.03 a	5.00 ± 0.58 c	136.0 ± 0.61 b	151.0 ± 1.02 a
Hydropriming	Control	86.25 ± 0.48 c	6.75 ± 0.48 a	123.0 ± 1.08 c	132.0 ± 0.35 c
	Bac	92.00 ± 0.75 a	6.75 ± 0.48 a	141.3 ± 0.20 b	157.0 ± 0.66 a
	Bj	87.75 ± 0.71 bc	7.25 ± 0.48 a	145.0 ± 1.69 a	155.1 ± 0.94 b
	Bac + Bj	90.00 ± 1.29 ab	7.00 ± 0.41 a	147.8 ± 0.59 a	149.1 ± 0.52 b
Zn priming	Control	87.50 ± 0.96 c	7.00 ± 0.41 a	126.1 ± 1.04 b	135.4 ± 1.25 c
	Bac	94.25 ± 0.41 a	6.25 ± 0.25 bc	152.9 ± 0.64 a	163.5 ± 0.72 a
	Bj	92.00 ± 1.03 ab	7.75 ± 0.63 ab	149.7 ± 0.55 a	152.9 ± 0.61 b
	Bac + Bj	90.74 ± 0.85 b	4.75 ± 0.48 c	151.8 ± 1.51 a	151.6 ± 0.52 b

Data are presented as means (*n* = 4) ± SE. Different letters in the same column denote statistically significant differences (*p* ≤ 0.05, Duncan’s multiple range test). Note: Bac, *Bacillus megaterium*; Bj, *Bradyrhizobium japonicum*; Bac + Bj, *Bacillus megaterium* and *Bradyrhizobium japonicum*.

**Table 3:** The effects of priming treatments on the fresh and dry shoot and root weight and seedling vigor index of soybean in the germination test.

Nutrient Priming	Biopriming	Fresh Shoot Weight (g)	Fresh Root Weight (g)	Dry Shoot Weight (g)	Dry Root Weight (g)	Seedling Vigor Index
Control	Control	8.24 ± 0.055 d	1.30 ± 0.006 d	0.736 ± 0.004 c	0.125 ± 0.001 d	2098.8 ± 31.6 d
	Bac	9.72 ± 0.019 c	1.57 ± 0.013 c	0.965 ± 0.004 a	0.151 ± 0.001 c	2481.1 ± 18.8 b
	Bj	10.15 ± 0.018 a	1.77 ± 0.014 a	0.969 ± 0.003 a	0.178 ± 0.000 a	2347.4 ± 36.3 c
	Bac + Bj	9.99 ± 0.039 b	1.62 ± 0.013 b	0.937 ± 0.001 b	0.154 ± 0.000 b	2604.9 ± 42.3 a
Hydropriming	Control	9.79 ± 0.065 b	1.89 ± 0.022 c	0.981 ± 0.010 a	0.163 ± 0.001 d	2199.5 ± 21.2 c
	Bac	9.88 ± 0.024 b	1.83 ± 0.030 c	0.981 ± 0.011 a	0.173 ± 0.001 c	2743.9 ± 22.3 a
	Bj	10.32 ± 0.028 a	2.30 ± 0.016 a	1.004 ± 0.003 a	0.182 ± 0.002 a	2633.6 ± 33.5 b
	Bac + Bj	10.21 ± 0.121 a	1.98 ± 0.023 b	1.007 ± 0.010 a	0.177 ± 0.001 b	2671.9 ± 38.1 b
Zn priming	Control	10.13 ± 0.180 b	2.06 ± 0.028 c	1.015 ± 0.017 c	0.197 ± 0.002 d	2287.2 ± 12.0 c
	Bac	10.33 ± 0.092 b	2.16 ± 0.019 c	1.082 ± 0.017 b	0.206 ± 0.002 c	2982.2 ± 19.1 a
	Bj	11.05 ± 0.049 a	2.50 ± 0.044 a	1.157 ± 0.003 a	0.241 ± 0.002 a	2783.8 ± 31.3 b
	Bac + Bj	11.07 ± 0.064 a	2.36 ± 0.045 b	1.169 ± 0.008 a	0.230 ± 0.002 b	2753.5 ± 34.2 b

Data are presented as means (*n* = 4) ± SE. Different letters in the same column denote statistically significant differences (*p* ≤ 0.05, Duncan’s multiple range test). Note: Bac, *Bacillus megaterium*; Bj, *Bradyrhizobium japonicum*; Bac + Bj, *Bacillus megaterium* and *Bradyrhizobium japonicum*.

**Table 4:** The effects of priming treatments, nutrient priming and biopriming, on seed germination, abnormal seedlings, and root and shoot length of soybean in the accelerated aging test.

Nutrient Priming	Biopriming	Seed Germination (%)	Abnormal Seedlings (%)	Shoot Length (mm)	Root Length (mm)
Control	Control	79.00 ± 0.41 b	12.25 ± 0.48 b	110.9 ± 0.31 d	74.5 ± 1.24 d
	Bac	83.50 ± 0.48 a	10.00 ± 0.48 c	148.6 ± 0.24 b	175.5 ± 0.24 a
	Bj	78.75 ± 0.29 b	16.25 ± 0.41 a	137.1 ± 0.73 c	142.1 ± 0.65 b
	Bac + Bj	84.75 ± 0.48 a	12.50 ± 0.50 b	150.5 ± 0.20 a	133.6 ± 0.24 c
Hydropriming	Control	79.50 ± 0.65 a	12.25 ± 1.03 a	93.25 ± 0.43 c	80.0 ± 0.20 d
	Bac	81.00 ± 0.29 a	12.25 ± 0.41 a	130.63 ± 0.41 a	132.4 ± 0.38 a
	Bj	76.50 ± 0.71 b	11.00 ± 0.63 a	119.0 ± 0.24 b	102.6 ± 0.43 c
	Bac + Bj	81.00 ± 0.71 a	10.25 ± 048 a	119.6 ± 0.83 b	104.5 ± 0.74 b
Zn priming	Control	80.25 ± 0.85 b	13.25 ± 1.80 a	107.6 ± 0.24 c	91.8 ± 0.32 b
	Bac	81.50 ± 0.65 ab	14.50 ± 1.44 a	140.8 ± 0.32 b	134.4 ± 0.78 a
	Bj	77.50 ± 0.65 c	12.50 ± 1.26 a	103.8 ± 0.32 d	73.8 ± 0.24 c
	Bac + Bj	84.00 ± 1.08 a	13.25 ± 0.41 a	148.3 ± 0.48 a	133.3 ± 1.16 a

Data are presented as means (*n* = 4) ± SE. Different letters in the same column denote statistically significant differences (*p* ≤ 0.05, Duncan’s multiple range test). Note: Bac, *Bacillus megaterium*; Bj, *Bradyrhizobium japonicum*; Bac + Bj, *Bacillus megaterium* and *Bradyrhizobium japonicum*.

**Table 5:** The effects of priming treatments, nutrient priming and biopriming, on the fresh and dry shoot and root weight and seedling vigor index of soybean in the accelerated aging test.

Nutrient Priming	Biopriming	Fresh Shoot Weight (g)	Fresh Root Weight (g)	Dry Shoot Weight (g)	Dry Root Weight (g)	Seedling Vigor Index
Control	Control	9.05 ± 0.201 b	1.13 ± 0.051 d	0.926 ± 0.028 a	0.125 ± 0.003 d	1464.5 ± 15.6 d
	Bac	8.99 ± 0.047 b	2.33 ± 0.009 a	0.847 ± 0.005 b	0.224 ± 0.001 a	2709.2 ± 15.4 a
	Bj	8.94 ± 0.027 b	1.88 ± 0.009 b	0.853 ± 0.002 b	0.188 ± 0.001 b	2199.1 ± 14.4 c
	Bac + Bj	9.80 ± 0.010 a	1.72 ± 0.010 c	0.944 ± 0.002 a	0.168 ± 0.001 c	2405.8 ± 13.5 b
Hydropriming	Control	9.84 ± 0.013 b	1.27 ± 0.009 c	1.018 ± 0.014 a	1.130 ± 0.001 d	1377.4 ± 12.8 d
	Bac	9.78 ± 0.013 b	1.70 ± 0.009 a	0.976 ± 0.006 b	0.166 ± 0.001 b	2130.4 ± 6.2 a
	Bj	9.98 ± 0.013 a	1.41 ± 0.017 b	1.019 ± 0.003 a	0.145 ± 0.001 c	1695.4 ± 21.4 c
	Bac + Bj	10.08 ± 0.033 a	1.69 ± 0.058 b	1.035 ± 0.018 a	0.177 ± 0.001 a	1813.4 ± 14.5 b
Zn priming	Control	9.38 ± 0.093 b	0.983 ± 0–005 c	0.912 ± 0.004 c	0.151 ± 0.002 b	1600.0 ± 18.6 c
	Bac	9.58 ± 0.033 b	1.763 ± 0.006 b	0.952 ± 0.004 b	0.175 ± 0.001 a	2242.2 ± 13.6 b
	Bj	10.58 ± 0.145 a	1.788 ± 0.014 ab	1.096 ± 0.011 a	0.171 ± 0.001 a	1375.7 ± 16.5 d
	Bac + Bj	9.01 ± 0.011 c	1.813 ± 0.011 a	0.880 ± 0.001 d	0.175 ± 0.000 a	2364.7 ± 33.4 a

Data are presented as means (*n* = 4) ± SE. Different letters in the same column denote statistically significant differences (*p* ≤ 0.05, Duncan’s multiple range test). Note: Bac, *Bacillus megaterium*; Bj, *Bradyrhizobium japonicum*; Bac + Bj, *Bacillus megaterium* and *Bradyrhizobium japonicum*.

**Table 6:** Pearson’s correlation of the examined seed quality parameters and growth-related parameters of soybean in the germination test (**a**) and accelerated aging test (**b**).

**(a) Germination Test**									
	**SG**	**AS**	**SL**	**RL**	**FSW**	**FRW**	**DSW**	**DRW**	**SVI**
SG	1.00	−0.39 **	0.69 ***	0.69 ***	0.49 ***	0.37 **	0.54 ***	0.44 **	0.87 ***
AS		1.00	−0.40 **	−0.36 *	−0.46 ***	−0.24 ^ns^	−0.47 ***	−0.26 ^ns^	−0.42 **
SL			1.00	0.80 ***	0.76 ***	0.69 ***	0.77 ***	0.70 ***	0.92 ***
RL				1.00	0.51 ***	0.57 ***	0.55 ***	0.52 ***	0.92 ***
FSW					1.00	0.87 ***	0.93 ***	0.88 ***	0.64 ***
FRW						1.000	0.88 ***	0.92 ***	0.61 ***
DSW							1.00	0.93 ***	0.69 ***
DRW								1.00	0.62 ***
SVI									1.00
**(b) Accelerated Aging Test**									
	**SG**	**AS**	**SL**	**RL**	**FSW**	**FRW**	**DSW**	**DRW**	**SVI**
SG	1.00	−0.08 ^ns^	0.66 ***	0.59 ***	−0.32 *	0.40 **	−0.45 ***	0.42 **	0.72 ***
AS		1.00	0.09 ^ns^	0.05 ^ns^	−0.25 ^ns^	−0.05 ^ns^	−0.21 ^ns^	−0.02 ^ns^	0.04 ^ns^
SL			1.00	0.90 ***	−0.45 ***	0.70 ***	−0.61 ***	0.69 ***	0.96 ***
RL				1.00	−0.48 ***	0.78 ***	−0.65 ***	0.82 ***	0.97 ***
FSW					1.00	−0.10 ^ns^	0.92 ***	−0.23 ***	−0.48 ***
FRW						1.00	−0.25 ^ns^	0.90 ***	0.76 ***
DSW							1.00	−0.40 **	−0.65 ***
DRW								1.00	0.77 ***
SVI									1.00

* *p* ≤ 0.05, ** *p* ≤ 0.01¸ *** *p* ≤ 0.001, ^ns^—non-significant. Note: SG, seed germination; AS, abnormal seedlings; SL, shoot length; RL, root length; FSW, fresh shoot weight; FRW, fresh root weight; DSW, dry shoot weight; DRW, dry root weight; SVI, seedling vigor index.

**Table 7:** The effects of priming treatments, nutrient priming and biopriming, on the occurrence of *Alternaria* spp. and *Fusarium* spp. on soybean seeds in the germination test.

Nutrient Priming	Biopriming	*Alternaria* spp. (%)	*Fusarium* spp. (%)
Control	Control	12.5 ± 0.3 a	8.5 ± 0.3 a
	Bac	3.5 ± 0.3 c	2.5 ± 0.3 b
	Bj	11.0 ± 0.4 b	9.0 ± 0.4 a
	Bac + Bj	5.0 ± 0.4 c	2.5 ± 0.3 b
Hydropriming	Control	12.0 ± 0.6 a	9.0 ± 0.4 a
	Bac	3.0 ± 0.4 c	2.0 ± 0.4 b
	Bj	10.0 ± 0.4 b	8.0 ± 0.6 a
	Bac + Bj	4.0 ± 0.4 c	2.0 ± 0.6 b
Zn priming	Control	7.0 ± 0.4 a	5.0 ± 0.0 a
	Bac	2.0 ± 0.4 b	1.0 ± 0.4 b
	Bj	6.0 ± 0.4 a	4.0 ± 0.4 a
	Bac + Bj	2.0 ± 0.0 b	0.75 ± 0.5 b

Data are presented as means (*n* = 4) ± SE. Different letters in the same column denote statistically significant differences (*p* ≤ 0.05, Duncan’s multiple range test). Note: Bac, *Bacillus megaterium*; Bj, *Bradyrhizobium japonicum*; Bac + Bj, *Bacillus megaterium* and *Bradyrhizobium japonicum*.

**Table 8:** The effects of priming treatments, nutrient priming and biopriming, on the occurrence of *Alternaria* spp. and *Fusarium* spp. on soybean seeds in the accelerated aging test.

Nutrient Priming	Biopriming	*Alternaria* spp. (%)	*Fusarium* spp. (%)
Control	Control	4.0 ± 0.4 a	4.0 ± 0.4 a
	Bac	3.0 ± 0.4 ab	2.5 ± 0.7 b
	Bj	3.75 ± 0.5 ab	1.5 ± 0.3 b
	Bac + Bj	2.0 ± 0.4 b	2.0 ± 0.4 b
Hydropriming	Control	4.0 ± 0.4 a	3.0 ± 0.4 a
	Bac	2.0 ± 0.4 b	1.0 ± 0.4 b
	Bj	4.0 ± 0.4 a	1.75 ± 0.3 ab
	Bac + Bj	2.0 ± 0.4 b	1.25 ± 0.3 b
Zn priming	Control	3.0 ± 0.4 a	2.0 ± 0.4 a
	Bac	1.5 ± 0.3 ab	0.75 ± 0.3 ab
	Bj	2.0 ± 0.4 ab	1.0 ± 0.4 ab
	Bac + Bj	1.0 ± 0.4 b	0.5 ± 0.3 b

Data are presented as means (*n* = 4) ± SE. Different letters in the same column denote statistically significant differences (*p* ≤ 0.05, Duncan’s multiple range test). Note: Bac, *Bacillus megaterium*; Bj, *Bradyrhizobium japonicum*; Bac + Bj, *Bacillus megaterium* and *Bradyrhizobium japonicum*.

## Data Availability

The data sets utilized and examined in the present study can be obtained from the corresponding author upon reasonable request.

## Supplementary Materials

The following supporting information can be downloaded at: https://www.mdpi.com/article/10.3390/plants13182557/s1. Table S1: Effect of seed nutrient priming and biopriming treatments on soybean parameters in germination test. Table S2: Effect of seed nutrient priming and biopriming treatments on soybean parameters in accelerated aging test. Table S3: Effect of seed nutrient priming and biopriming treatments on the occurrence of *Alternaria* spp. and *Fusarium* spp. on soybean seeds in germination and accelerated aging tests.
