# Supplementary material for: Impact of Simultaneous Nutrient Priming and Biopriming on Soybean Seed Quality and Health
Source: Plants (Basel). 2024 Sep 11;13(18):2557. doi: 10.3390/plants13182557 (PMC11434937; doi:10.3390/plants13182557)
Supplement: Supplementary file 1 [file plants-13-02557-s001.zip › plants-3200924-supplementary.pdf]

Supplementary Table S1. Effect of seed nutrient and biopriming treatments on soybean parameters in germination test.

Nutrient priming	Biopriming				
	Control	Bac	Bj	Bac+Bj	Average
**Seed Germination (%)**					
Control	84.75 e	91.00 b	86.50 de	90.75 b	88.25 B
HP	86.25 de	92.00 b	87.75 cd	90.00 b	89.00 B
ZnP	87.50 cde	94.25 a	87.75 cd	90.75 b	91.13 A
Average	86.17 D	92.42 A	88.75 C	90.50V B	89.46
**Abnormal Seedlings (%)**					
Control	9.50 a	6.25 bc	7.50 b	5.00 cd	7.06 A
HP	6.75 b	6.75 b	7.25 b	7.00 b	9.94 A
ZnP	7.00 b	6.25 bc	7.75 b	4.75 d	6.44 A
Average	7.75 A	6.42 B	7.50 A	5.58 C	7.81
**Shoot Length (mm)**					
Control	116.5 i	137.4 f	138.4 f	136.0 f	132.1 C
HP	123.0 h	141.3 e	145.0 d	147.8 c	139.3 B
ZnP	126.1 g	152.9 a	149.7 bc	151.8 ab	145.1 A
Average	121.9 B	143.9 A	144.4 A	145.2 A	138.8
**Root length (mm)**					
Control	131.1 g	135.3 f	133.0 fg	151.0 de	137.6 C
HP	132.0 g	157.0 b	155.1 bc	149.1 e	148.3 B
ZnP	135.4 f	163.5 a	152.9 d	151.6 d	150.8 A
Average	132.8 D	151.9 A	147.0 C	150.6 B	145.6
**Fresh shoot weight (g)**					
Control	8.240 f	9.720 e	10.150 bc	9.988 cd	9.524 C
HP	9.878de	9.790 de	10.323 b	10.208 bc	10.049 B
ZnP	10.128 bc	10.334 b	11.048 a	11.065 a	10.643 A
Average	9.415 C	9.948 B	10.507 A	10.420 A	10.072
**Fresh Root Weight (g)**					
Control	1.303 h	1.572 g	1.768 f	1.623 g	1.566 C
HP	1.890 e	1.830 fe	2.298 b	1.988 d	2.001 B
ZnP	2.060 d	2.163 c	2.498 a	2.358 b	2.269 A
Average	1.751 D	1.855 C	2.188 A	1.989 B	1.945
**Dry Shoot Weight (g)**					
Control	0.736 g	0.965 e	0.969 e	0,937 f	0.902 C
HP	0.981 de	0.981 de	1.004 cd	1.007 cd	0.993 B
ZnP	1.015 c	1.082 b	1.157 a	1.169 a	1.106 A
Average	0.911 C	1.009 B	1.043 A	1.038 A	1.000
**Dry Root Weight (g)**					
Control	0.125 j	0.151 i	0.178 fe	0.154 i	0.152 C
HP	0.163 h	0.173 g	0.182 e	0.177 fg	0.174 B
ZnP	0.197 d	0.206 b	0.241a	0.230 b	0.218 A
Average	0.162 D	0.177 C	0.200 A	0.187 B	0.181

**Seedling Vigor Index**					
Control	2098.8 h	2481.1 e	2347.4 f	2604.9 d	2383.1 C
HP	2199.5 g	2743.9 bc	2633.6 d	2671.cd	2562.2 B
ZnP	2287.2 f	2982.2 a	2783.8 b	2753.5 bc	2701.7 A
Average	2195.2 D	2735.8 A	2588.2 C	2676.8 B	2549.0

Data are represented as mean (n=4); Differences between treatments were analysed using Duncan's multiple range test ($p \leq 0.05$). Means within each trait followed by the same lowercase/uppercase letters are not significantly different. Note: Bac, *Bacillus megaterium*; Bj, *Bradyrhizobium japonicum*; Bac+Bj, *Bacillus megaterium* and *Bradyrhizobium japonicum*.

Supplementary Table S2. Effect of seed nutrient and biopriming treatments on soybean parameters in accelerated aging test

Nutrient priming	Biopriming				
	**Control**	**Bac**	**Bj**	**Bac+Bj**	**Average**
**Seed Germination (%)**					
Control	79.0 cde	83.5 a	78.75 de	84.75 a	81.5 A
HP	79.5 bcd	81.00bc	76.50 f	81.00bc	79.50 B
ZnP	80.25 bcd	81.50 b	77.50 ef	84.00 b	80.81 A
Average	79.58 C	82.00 B	77.58 D	83.25 A	80.60
**Abnormal Seedlings (%)**					
Control	12.25 bcd	10.00 d	16.25 a	12.50 bcd	12.75 A
HP	12.25 bcd	12.25 bcd	11.00 cd	10.25 cd	11.44 B
ZnP	13.25 bc	14.50 ab	12.50 bcd	13.00bcd	13.31 A
Average	12.58 A	12.25 A	13.25 A	11.92 A	12.50
**Shoot Length (mm)**					
Control	110.88 g	148.60 b	137.13 d	150.50 a	136.77 A
HP	93.25 j	130.63 e	119.00 f	119.38 f	115.56 C
ZnP	107.63 h	140.75 c	103.75 i	148.25 b	125.09 B
Average	103.92 C	139.99 A	119.96 B	139.38 A	125.81
**Root length (mm)**					
Control	74.50 h	175.50 a	142.13 b	133.38 c	131.38 A
HP	80.00 g	132.38 c	102.63 e	104.50 d	104.88 B
ZnP	91.75 e	134.38 c	73.75 h	133.25 c	108.28 C
Average	82.08 D	147.42 A	106.17 C	123.71 B	114.85
**Fresh shoot weight (g)**					
Control	9.053 f	8.990 f	8.942 f	9.802 cd	9.197 C
HP	9.838 bc	9.783 cd	9.982 bc	10.082 b	9.921 A
ZnP	9.382 e	9.575 de	10.582 a	9.010 f	9.638 B
Average	9.424 C	9.449 C	9.836 A	9.632 B	9.585
**Fresh Root Weight (g)**					
Control	1.130 g	2.328 a	1.878 b	1.715 d	1.763 A
HP	1.268 e	1.703 d	1.410 e	1.693 d	1.518 C
ZnP	0.983 h	1.762 cd	1.788 c	1.813bc	1.586 B

Average	1.127 D	1.931 A	1.692 C	1.740 B	1.623
**Dry Shoot Weight (g)**					
Control	0.926 de	0.847 g	0.853 g	0.944 cde	0.893 C
HP	1.018 b	0.976 c	1.019b	1.035 b	1.012 A
ZnP	0.912 e	0.951 cd	1.096 a	0.944 cde	0.960 B
Average	0.952 B	0.925 C	0.989 A	0.953 B	0.955
**Dry Root Weight (g)**					
Control	0.125 i	0.224 a	0.188 b	0.168 e	0.176 A
HP	0.129 h	0.166 e	0.145 g	0.177 c	0.154 C
ZnP	0.151 f	0.175 cd	0.171 d	0.175 cd	0.168 B
Average	0.135 D	0.188 A	0.168 C	0.173 B	0.166
**Seedling Vigor Index**					
Control	1464.5 h	2706.2 a	2199.1 c	2405.8 b	2193.9 A
HP	1377.3 i	2130.3 d	1695.4 f	1913.4 d	1754.1 C
ZnP	1600.0 g	2242.2 c	1375.7 i	2364.7 b	1895.7 B
Average	1480.6 D	2359.6 A	1756.7 C	2194.6 B	1947.7

Data are represented as mean (n=4); Differences between treatments were analysed using Duncan's multiple range test ($p \leq 0.05$). Means within each trait followed by the same lowercase/uppercase letters are not significantly different. Note: Bac, *Bacillus megaterium*; Bj, *Bradyrhizobium japonicum*; Bac+Bj, *Bacillus megaterium* and *Bradyrhizobium japonicum*.

Supplementary Table S3. Effect of seed nutrient and biopriming treatments on the occurrence of *Alternaria* spp. and *Fusarium* spp. on soybean seeds in germination and accelerated aging tests.

**a) Germination Test**					
**Nutrient priming**	**Biopriming**				
	**Control**	**Bac**	**Bj**	**Bac+Bj**	**Average**
**Alternaria spp. (%)**					
Control	13.0 a	3.5 efg	11.0 b	5.0 de	7.00 A
HP	12.0 a	3.0 fg	10.0 b	4.0 fe	6.00A
ZnP	7.0 c	2.0 g	6.0 cd	2.0 g	4.00B
Average	10.00 A	3.00 C	8.00 B	4.00 C	6.00
**Fusarium spp. (%)**					
Control	8.5 a	2.5 a	9.0 cd	2.5 cd	5.63A
HP	9.0 a	8.0 a	2.0 de	2.0 de	5.25A
ZnP	5.0 b	1.0 ef	4.0 bc	0.75 f	2.69B
Average	7.50A	1.83B	7.00A	1.75B	4.52
**b) Accelerated Aging Test**					
**Alternaria spp. (%)**					
Control	4.0 a	3.0 ab	3.75 a	2.0 bc	3.19A
HP	4.0 a	4.0 a	2.0 bc	2.0 bc	3.00A

ZnP	3.0 ab	1.5 bc	2.0 bc	1.0 c	1.88B
Average	3.67A	2.17B	3.25A	1.67B	2.69
**Fusarium spp. (%)**					
Control	4.0 a	1.5 bcde	2.5 bc	2.0 bcd	2.50A
HP	3.0 a	1.0de	1.75 bcd	1.25 cde	1.75B
ZnP	2.0 bcd	0.75 de	1.0 de	0.5 e	1.06C
Average	3A	1.08B	1.75B	1.25B	1.77

Data are represented as mean (n=4); Differences between treatments were analysed using Duncan's multiple range test ($p \leq 0.05$). Means within each trait followed by the same lowercase/uppercase letters are not significantly different. Note: Bac, *Bacillus megaterium*; Bj, *Bradyrhizobium japonicum*; Bac+Bj, *Bacillus megaterium* and *Bradyrhizobium japonicum*.
